# Dengue virus antibody database: Systematically linking serotype-specificity with epitope mapping in dengue virus

**DOI:** 10.1371/journal.pntd.0005395

**Published:** 2017-02-21

**Authors:** Sidhartha Chaudhury, Gregory D. Gromowski, Daniel R. Ripoll, Ilja V. Khavrutskii, Valmik Desai, Anders Wallqvist

**Affiliations:** 1 Biotechnology HPC Software Applications Institute, Telemedicine and Advanced Technology Research Center, U.S. Army Medical Research and Materiel Command, Fort Detrick, Maryland, United States of America; 2 Viral Diseases Branch, Walter Reed Army Institute of Research, Silver Spring, Maryland, United States of America; Oregon Health and Science University, UNITED STATES

## Abstract

**Background:**

A majority infections caused by dengue virus (DENV) are asymptomatic, but a higher incidence of severe illness, such as dengue hemorrhagic fever, is associated with secondary infections, suggesting that pre-existing immunity plays a central role in dengue pathogenesis. Primary infections are typically associated with a largely serotype-specific antibody response, while secondary infections show a shift to a broadly cross-reactive antibody response.

**Methods/Principal findings:**

We hypothesized that the basis for the shift in serotype-specificity between primary and secondary infections can be found in a change in the antibody fine-specificity. To investigate the link between epitope- and serotype-specificity, we assembled the Dengue Virus Antibody Database, an online repository containing over 400 DENV-specific mAbs, each annotated with information on *1*) its origin, including the immunogen, host immune history, and selection methods, *2*) binding/neutralization data against all four DENV serotypes, and *3*) epitope mapping at the domain or residue level to the DENV E protein. We combined epitope mapping and activity information to determine a residue-level index of epitope propensity and cross-reactivity and generated detailed composite epitope maps of primary and secondary antibody responses. We found differing patterns of epitope-specificity between primary and secondary infections, where secondary responses target a distinct subset of epitopes found in the primary response. We found that secondary infections were marked with an enhanced response to cross-reactive epitopes, such as the fusion-loop and E-dimer region, as well as increased cross-reactivity in what are typically more serotype-specific epitope regions, such as the domain I-II interface and domain III.

**Conclusions/Significance:**

Our results support the theory that pre-existing cross-reactive memory B cells form the basis for the secondary antibody response, resulting in a broadening of the response in terms of cross-reactivity, and a focusing of the response to a subset of epitopes, including some, such as the fusion-loop region, that are implicated in poor neutralization and antibody-dependent enhancement of infection.

## Author summary

Dengue virus (DENV) infections are typically asymptomatic, but severe and potentially lethal disease symptoms, such as dengue hemorrhagic fever, are associated with secondary infections. This suggests that pre-existing immunity from primary infection plays a central role in DENV pathogenesis. In order to characterize the antibody response in primary and secondary infections, we assembled the Dengue Virus Antibody Database, a freely accessible online repository (http://denvabdb.bhsai.org) storing over 400 unique monoclonal dengue-specific antibodies annotated by their 1) origin and host immune history, 2) activity information against all four dengue serotypes, and 3) epitope mapping information. Here we demonstrate the utility of the database by carrying out a large-scale analysis to characterize shifts in epitope fine-specificity and serotype cross-reactivity in primary and secondary infections. In particular, we show how the antibody response in secondary infections displays a systematic shift towards increased serotype cross-reactivity by focusing on a subset of cross-reactive epitopes on the dengue E protein. Our findings suggest a mechanistic basis for this shift in epitope and serotype specificity and demonstrate how a detailed understanding of the antibody response can provide insight into the mechanisms of dengue pathogenesis.

## Introduction

Dengue virus (DENV), an arthropod-borne virus of the *Flaviviridae* family, infects an estimated 400 million people each year [1]. There are four antigenically related DENV serotypes, DENV 1–4, each capable of causing disease. DENV infections are often asymptomatic or result in an uncomplicated fever and can elicit life-long immunity to the infecting serotype and short-term cross-protection against heterotypic DENV infections [2–5]. Although, recent studies have demonstrated that homotypic DENV reinfection is possible [6]. Secondary infection with a heterotypic DENV serotype results in a higher incidence of more severe disease and cross-reactive antibodies are thought to contribute to this by a mechanism termed antibody-dependent enhancement (ADE) of infection [7–11]. The antibody response following secondary infection is broadly cross-reactive among DENV serotypes and longer periods of cross-protection are observed [3, 12]. Further characterizing differences in the antibody response to primary and secondary heterotypic DENV infections, and how these differences are associated with serotype-specificity and neutralization, is critical to understanding DHF pathogenesis and developing dengue vaccines.

The DENV virion consists of 180 copies of the envelope (E) protein, arranged in 90 dimers in an icosahedral ‘herring-bone’ geometry [13] and is the primary target of DENV neutralizing antibodies [14]. The soluble portion of the E protein consists of three distinct domains [15], termed Domain I (DI), Domain II (DII), and Domain III (DIII). Neutralizing antibodies (Abs) targeting E, reviewed in [16], are the main focus of current DENV vaccine development efforts. Not all E protein-specific Abs contribute equally to virus neutralization and neutralizing Ab potency is related to its epitope. Early work with mouse mAbs indicated that DIII was a major target of potently neutralizing DENV mAbs [17–27]. However, a low fraction of DIII-specific neutralizing Abs are found in human sera post-DENV infection and they only appear to make a minor contribution to DENV neutralization [28–31]. The human neutralizing Ab response appears to preferentially target the DI/DII hinge region of E protein monomers [32–34] and quaternary E protein epitopes that are only present in the context of intact virions [32, 35, 36]. Finally, DENV Abs can vary with respect to serotype cross-reactivity. Complex Abs bind or neutralize all four serotypes, type-specific Abs bind or neutralize only a single serotype, and sub-complex Abs bind or neutralize more than one, but not all four serotypes. It is important to note that there are significant strain and genotype-level differences in antibody neutralization within a serotype as well [27, 37–39].

The antibody response to dengue infection is a polyclonal response that is thought to consists of a repertoire of >10^3^ unique monoclonal antibodies (mAbs) [40]. Previous studies from polyclonal sera [41, 42], and panels of monoclonal antibodies [36, 43–45], have shown that the serotype-specificity of the antibody response shifts between primary and secondary infections. Primary infections are characterized by a largely type-specific antibody response while secondary infections result in a broadly cross-reactive response.

We hypothesize that the basis for the shift in serotype-specificity between primary and secondary antibody responses can be found in a change in the fine-specificity—the relative response to different epitopes on the E protein. To investigate the link between epitope fine-specificity and serotype-specificity, we assembled the Dengue Virus Antibody Database (http://denvabdb.bhsai.org), an online repository containing 410 DENV-specific mAbs, each annotated with information on *1*) its origin, including the immunogen, host immune history, and selection methods, *2*) binding or neutralization data against four DENV serotypes, and *3*) epitope mapping at the domain or residue level. Because the database contains information linking infection type (primary vs. secondary) and serotype-specificity (type-specific, sub-complex, complex), with residue or domain-level epitope mapping, it allows us to identify the epitope-level determinants of observed shifts in type-specificity associated with secondary infections. While analysis of any single study or panel of DENV mabs may reveal only a limited understanding of the polyclonal diversity of the DENV antibody response, we hypothesize that a large-scale analysis of hundreds of mAbs collected across dozens of diverse studies may be able to identify systematic trends in the DENV antibody repertoire and provide key insights into DENV antibody cross-reactivity and fine specificity. Finally, it is important to note that this study represents a meta-analysis of decades of previous studies on dengue mAbs, and as such seeks to provide quantitative basis for previously observed differences in cross-reactivity and fine-specificity in antibody responses to primary and secondary DENV infections.

## Materials and methods

### Database composition

We assembled a database of DENV mAbs described in literature that fulfill the following criteria: *1*) has information on how the mAb was isolated, in terms of the immunogen, the host organism, and immune history; *2*) has *in vitro* binding or neutralization data against all four DENV serotypes; and *3*) binds to E protein, and has epitope mapping information with at least a domain-level resolution. Overall, we found 410 mAbs that matched these criteria.

**Fig 1:**
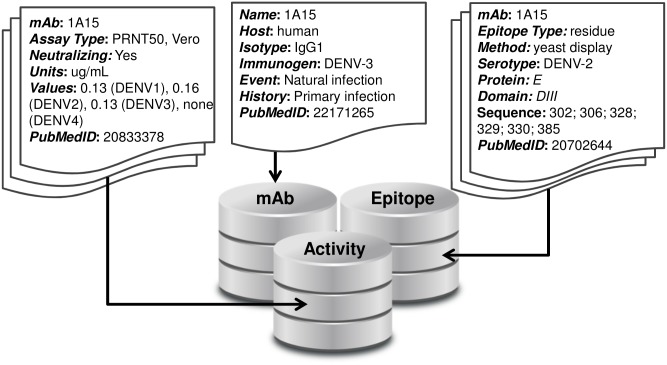
Dengue virus antibody database. Each monoclonal antibody in the database is annotated with information about its origin and selection, activity against all four dengue serotypes, epitope mapping, and relevant references.

There are three linked sections of the database: mAb, Activity, and Epitope (Fig 1). The mAb section contains a single record for each mAb in the database that includes the mAb name and information about its origin, including the host organism, the isotype, the immunogen, the exposure event that lead to the antibody, the host immune history, the selection criteria used to isolate or select that mAb for study, and the PubMedID of the reference that first reported the mAb. The Activity section can contain multiple records for each mAb and includes information on the activity assay details and data against all four DENV serotypes, and the corresponding PubMedID for its reference. This data could be qualitative, such as in the case of a Western Blot, or quantitative, as in the case of titers from a neutralization assay. The Epitope section also contains multiple records for each mAb and includes information on the epitope resolution or type (‘residue’ or ‘domain’), the epitope mapping method, the domain of E that the mAb was mapped to, the E amino acid sequence that describes that epitope, and the PubMedID of the corresponding reference. The Dengue Antibody Database is freely accessible online at http://denvabdb.bhsai.org. Through the web-based interface, users are able to search for particular mAbs, sort mAbs by various properties, and download any information on the database directly as a spreadsheet.

### Antibody serotype-specificity classification

Based on the available activity information, we classified the serotype-specificity of each mAb in the database as ‘type,’ ‘sub-complex,’ and ‘complex’ based on the number of serotypes it was reactive towards. In the instance that different assays for the same mAb showed differing patterns of specificity, we classified the mAb based on the most serotype-restricted assay result. Among the cases where assays had conflicting serotype-specificity results, we found that, in general, neutralization assays tended to be more serotype-restricted than binding assays. We treated all activity methods equally to maximize the sample size for our analyses, however users are able to download the activity data from the database in spreadsheet form, and select subsets of mAbs that have data from certain activity methods. Finally, some studies also identify group-specific mAbs, antibodies that can bind to multiple different flaviviruses. However, since such characterization is available for only a limited number of mAbs in the database, we restricted our study to type, sub-complex, and complex mAbs. For many of the measurements, we focused our analysis on comparing type and complex mAbs. This was for two reasons: 1) the number of sub-complex mAbs was insufficient to generate statistically significant results for many measures, and 2) we found that sub-complex mAbs, in particular, were sensitive to assay-level variation in classification of their type-specificity, meaning they could not always be reliably distinguished from complex mAbs.

### Determining epitope propensity

We developed a measure of epitope-level antigenicity to describe a set of mAbs in terms of ‘epitope propensity,’ or the probability that a given E protein residue is found within the epitope definitions that make up the set of mAbs. Briefly, a set of mAbs consists of *N*_*r*_ mAbs with residue-level epitope definitions and *N*_*d*_ mAbs with domain-level epitope definitions. Note that the same mAb may have both residue and domain-level definitions. We define the residue-level epitope definition of mAb *j*, as a set of residues, $E_{j}^{res}=\left\{x_{1},x_{2},x_{3}\ldots x_{n}\right\}$ and the domain-level epitope definition of mAb *j* as $E_{j}^{dom}="DI/DII"$ or "*DIII*". *Q*(*i*) is the number of times residue *i* is found among all *N*_*r*_ residue-level epitope definitions in the data set (Eq 1):

$$Q\left(i\right)=\;\sum_{j}^{N_{r}}N\left(i\in E_{j}^{res}\right)$$ (1)

The epitope propensity at residue *i*, *P*(*i*), defined in Eq 2, is the probability of finding residue *i* among the *N*_*r*_ residue-level epitopes that lie within the domain of residue *i* (*d*_*i*_), multiplied by the probability of finding a epitope in *d*_*i*_, as determined by *N*_*d*_ domain-level epitope definitions.

$$P\left(i\right)=P\text{(}i\;|\;d_{i})\;\cdot \;P\left(d_{i}\right)$$ (2)

*P*(*i* | *d*_*i*_) is calculated by dividing the total count of residue *i*, by the total count across all epitope residues that fall within the domain *d*_*i*_, as shown in Eq 3. *P*(*d*_*i*_) is determined by the total count of domain level epitopes that are the same domain as *d*_*i*_ divided by the total number of domain-level epitopes in the data set, as shown in Eq 4.

$$P\text{(}i\;|\;d_{i})=Q\left(i\right)\;\cdot \;{\left(\sum_{k}^{\left|D_{i}\right|}Q\left(k\right)\right)}^{-1}$$ (3)

$$P\left(d_{i}\right)=\;\sum_{j}^{N_{d}}N\left(d_{i}\in E_{j}^{dom}\right)\;\cdot \;{\left(N_{d}\right)}^{-1}$$ (4)

By defining epitope propensity as a function of *P*(*i* | *d*_*i*_) and *P*(*d*_*i*_) we can use the residue-level epitope definition to determine the high-resolution details of mAbs in the database, while using domain-level epitope definitions to determine the relative immunogenicity of larger segments of the antigen. In any empirical measure there is the risk of observation bias—that researchers intentionally studying antibodies to a particular epitope region will bias a propensity measure calculated from those observations. By including a separate domain-level term, we can, to some degree, account for this potential observation bias.

### Calculating epitope-level cross reactivity

In addition to the epitope-level measure of propensity, we calculated aggregate epitope-level cross-reactivity from mAbs derived from primary and secondary infection. For each residue, we identified every mAb within the mAb subset (primary or secondary infection) that listed that residue as an epitope. We then determined the percentage of mAbs associated with that epitope residue that were classified as ‘complex’. If a residue had 60% or greater cross-reactivity, meaning at least 60% of all mAbs associated with that residue were classified as ‘complex’, we defined it as being of ‘high’ cross-reactivity. Residues with cross reactivity 30–60% cross-reactivity were defined as having ‘medium’ cross-reactivity; residues with <30% cross-reactivity were defined as having ‘low’ cross-reactivity.

### Measuring sequence variation in DENV epitopes

We carried out two separate, but related, analyses to assess the degree of sequence variation in DENV epitopes on E: a residue-level measure of sequence conservation and an epitope-level measure of antigenic mismatch, known as *p*_epitope_ [46, 47]. We carried out multiple sequence alignment using a set of 47 sequences of DENV across all four serotypes that were studied by Katzelnick *et al*. [37] for serum cross-reactivity and neutralization. We downloaded the E protein sequences for each strain from Genbank (see S2 Table). We then carried out a structure-based multiple sequence alignment using the Consurf algorithm [48] using the crystal structure of the DENV E protein [15] (PDB code: 1OKE). We defined sequence conservation at each residue position as the percentage of sequences that had the most common amino acid at that position.

$$p_{epitope}\left(S1,\;S2,\;mAb\;j\right)=\;\frac{\#\;of\;mismatches\;between\;S1\;and\;S2\;along\;E_{j}^{res}}{\left|E_{j}^{res}\right|}$$ (5)

*p*_*epitope*_ is an epitope-level measure of antigenic mismatch between two viral strains and can be used to predict the likelihood that immunity to one strain would provide protection against the other. We used one representative strain of DENV for each of the four serotypes, which was the consensus sequence for the E protein for each of the four serotypes as determined by a previous bioinformatics study by Danecek *et al*. [49]. For a residue-level epitope definition for mAb *j*, $E_{j}^{res}$, *p*_*epitope*_ is calculated by dividing the number of mismatches between two strains (S1 and S2) along the residues defined by $E_{j}^{res}$ with the total number of residues that defines $E_{j}^{res}$—describing a mismatch percentage along a defined set of epitope residues (Eq 5). We used ClustalW [50] to carry out the alignment and defined a mismatch as identified non-conserved or semi-conserved substitutions (denoted by ‘ ‘ and ‘.’, respectively, in the ClustalW sequence alignment file) in the alignment. For each mAb in the dataset with residue-level epitope definitions of five residues or more, we calculated *p*_*epitope*_ for all pairwise comparisons between the four serotypes and report the average *p*_*epitope*_ value as the *p*_*epitope*_ for that mAb.

In addition to calculating *p*_*epitope*_ values between representative sequences of DENV1-4, we also calculated pairwise *p*_*epitope*_ values for each strain within each serotype for the 47 sequences in the Kaetzelnick data set in order to compare intra-serotype and inter-serotype sequence variation at the epitope level.

## Results

### Database composition

There are three linked sections of the database: mAb, Activity, and Epitope (Fig 1). The mAb section contains a single record for each mAb in the database that includes the mAb name and information about its origin, including the host organism, the isotype, the immunogen, the exposure event that lead to the antibody, the host immune history, the selection criteria used to isolate or select that mAb for study, and the PubMedID of the reference that first reported the mAb. The Activity section can contain multiple records for each mAb and includes information on the activity assay details and data against all four DENV serotypes, and the corresponding PubMedID for its reference.

The breakdown of the database is shown in Table 1. Overall, the database is approximately evenly divided between mouse mAbs and human mAbs, from both primary and secondary infection. ELISA and neutralization assays are the most common activity records, accounting for almost 75% of the activity information. In Epitope records, there are only eight cryo-EM structures and eighteen x-ray crystallographic structures, representing <4% of the mAbs in the database which reflects the relative paucity of high-resolution epitope information on DENV mAbs. Most mAbs in the database have either mutagenesis or yeast-display data, underscoring the value of synthesizing this sparse epitope information to build a more comprehensive picture of DENV E protein epitopes.

**Table 1:** Database composition.

***Host (Infection type)***	***# of mAbs***
Mouse	172
Human (primary)	99
Human (secondary)	138
***Activity Method***	***# of records***
IFA	41
ELISA	253
ELISPOT	18
Neutralization	163
Flow cytometry	87
IP	23
Western blot	10
***Epitope Mapping Method***	***# of records***
X-ray crystallography	18
Cryo-EM	8
Mutagenesis	264
Yeast display	184
PepScan	27
Passaging	11
Western Blot	30

It is important to note that factors such as the antigen used for B cell selection, and even the time point, post-infection, when cell samples were collected to isolate antigen-specific B cells, can play a major role in epitope fine-specificity and cross-reactivity. We analyzed the database for all human antibodies, and for human and mouse antibodies, irrespective of these factors, in order to maximize the sample size for the analysis. However, this information is included in the database, and attached in S2 Table, for use in any further study. In certain cases, outlined in their respective sections, we did look for biases that selection methods may have had on the results.

### Serotype-specificity in primary and secondary dengue infections

**Fig 2:**
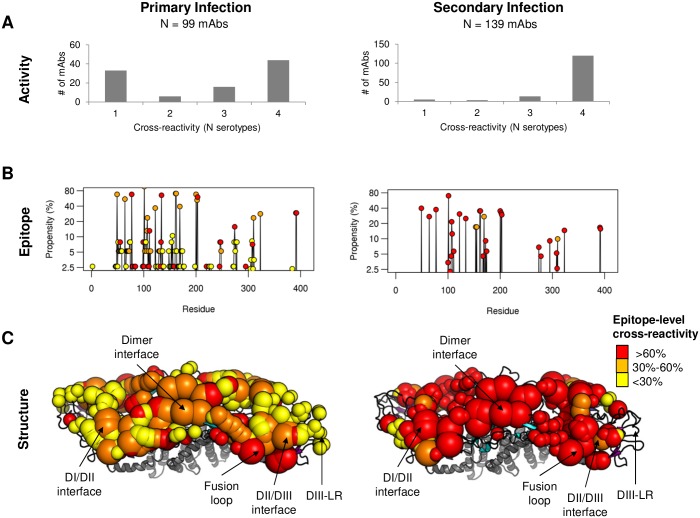
Serotype-specificity and epitope mapping in primary and secondary dengue infections. A) Histograms of human mAbs in the primary (left) and secondary infection (right) data sets. B) Epitope propensity in primary (left) and secondary (right) infections across E protein amino acid sequence. Points are colored with respect to epitope cross-reactivity, or the proportion of mAbs that are associated with a given epitope residue that are classified as ‘complex’: red (>60%), orange (30–60%), or yellow (< 30%). C) A composite map of epitope propensity and cross-reactivity on the structure of the DENV E protein dimer for mAbs from primary (left) and secondary (right) infections. Epitope regions DI/DII interface, dimer interface, fusion loop, DII/DIII interface, and DIII-lateral ridge (LR) are highlighted. Spheres correspond to epitope residues. The size of the sphere corresponds to its epitope propensity: low propensity (<5%, small spheres), medium propensity (>5% and <10%; medium spheres), and high propensity (>10%, large spheres). The color of the sphere corresponds to the epitope cross-reactivity as described above.

Overall, 45% of published mAbs from human primary infection were type-specific, 21% were sub-complex, and 34% were complex, while for mAbs from human secondary infection, 4% were type-specific, 10% were sub-complex, and 86% were complex (Fig 2a). These results reflect findings from polyclonal sera reported elsewhere [41] and underscore the profound shift from a type-specific primary immune response to an almost entirely cross-reactive secondary immune response. We next sought to map the epitopes from primary and secondary responses and determine the link between the epitope fine-specificity of the antibody response and its serotype-specificity.

### Mapping fine-specificity and cross-reactivity at the epitope level

We calculated epitope propensity across all residues in the DENV E protein from published human mAbs from primary and secondary DENV infections (Fig 2b). Overall, the results show that mAbs from secondary infection bind to a subset of the epitope residues for mAbs from primary infections. We also calculated epitope cross-reactivity as the percentage of mAbs associated with a particular epitope residue that have a serotype-specificity classification of ‘complex.’ We found that epitope residues from primary infections had a mix of cross-reactivity, ranging from low (<30%), medium (30%-60%), and high (>60%), while epitope residues from secondary infections were almost entirely of high cross-reactivity.

We generated a ‘composite’ epitope map of DENV antibody responses by mapping the epitope propensity and cross-reactivity of human mAbs from primary and secondary infections to the structure of the DENV E protein dimer [15] (Fig 2c). We found three overall trends. First, the fusion loop region, which showed high cross-reactivity in both primary and secondary responses, show an enhanced immunogenicity in secondary infections. Second, the DIII region, which displayed comparable immunogenicity in primary and secondary infections, showed a marked shift in both cross-reactivity and fine-specificity. The DIII response in secondary infections, unlike in primary infections, was highly cross-reactive, and shifted away from the lateral-ridge epitopes and towards the A-strand and DII/DIII interface epitopes. Finally, we found that two other epitope regions, the dimer interface, and the DI/DII interface, showed moderate immunogenicity in primary infections, with a medium level of cross-reactivity. In secondary infections, both of these regions showed a shift to high cross-reactivity.

### Sequence variation and serotype cross-reactivity

Katzelnick et al. found that although DENV strains cluster into discrete serotypes with respect to sequence similarity within the E protein, they do not cluster into discrete serotypes in terms of antigenic similarity [37]. We hypothesized that, among epitope residues identified in the database, the concordance between antigenic and sequence similarity might be greater. We used the set of 47 DENV strains studied by Katzelnick *et al*., to quantify the degree of sequence variation between the four serotypes to determine if epitopes associated with type-specific mAbs could be distinguished from epitopes associated with cross-reactive mAbs. We carried out a multiple sequence alignment using the Consurf algorithm [48] to align E protein sequences from all 47 DENV strains. For each residue in E, we calculated a sequence conservation measure as the percentage of the aligned sequences that had the most common amino acid at that position. In S1 Fig, we show the sequence diversity (defined as 1 − *sequence conservation*), across the E protein for all four serotypes. It is important to note that we sought specifically to compare intra-serotype variation from our epitope-based analysis with the results from the Katzelnick study, not characterize intra-serotype variation more generally.

**Fig 3:**
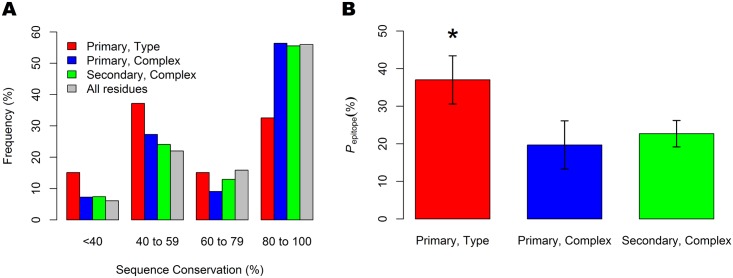
Sequence variation and serotype specificity in DENV mAbs. A) Histogram of sequence conservation among DENV 1–4 E protein epitope residues for type-specific and complex mAbs from primary and secondary infections, compared with all residues in the E protein. B) Sequence variation between consensus sequences of DENV1-4, at the epitope level measured by the average *p*_*epitope*_ for type-specific mAbs from primary infection (*N* = 45), complex mAbs from primary infection (*N* = 24), and complex mAbs from secondary infections (*N* = 25). Only mAbs with epitope definitions consisting of at least five residues were considered. Error bars correspond to the standard deviation of *p*_*epitope*_ values. A statistically significant difference is indicated by ‘*’, corresponding to p < 0.001.

We generated histograms with respect to sequence conservation among DENV 1–4 E protein epitope residues from type-specific and complex mAbs from primary and secondary infections (Fig 3A). Our results show that epitope residues for type-specific mAbs show a bimodal distribution with a majority of residues falling between 40% to 80% sequence conservation. By contrast, most epitope residues from complex-specific mAbs fall in the 80% to 100% sequence conservation range. For comparison, the sequence conservation for all residues shows that distribution if the respective type-specific and complex antibody responses were to target residues at random. These results show that primary type-specific antibodies preferentially target residues with lower sequence conservation. Furthermore, they show that complex antibodies do not have strong preference for conserved residues, as might be expected.

Previous research in antigenic variation in influenza virus identified a sequence-based measure, *p*_*epitope*_ that was found to be correlated with antibody cross-neutralization [46, 47] between strains. *p*_*epitope*_ is calculated by determining the proportion of sequence mismatches across a defined epitope region, between two strains. In order to calculate a reliable *p*_*epitope*_ value, an epitope definition of a sufficient size is necessary. For each mAb in the database with a residue-level epitope definition of at least five residues, we calculated a *p*_*epitope*_ value for that epitope based on the average pairwise *p*_*epitope*_ across all possible pairs of the four DENV serotypes (Fig 3B). For reference, a distribution of defined epitope sizes is shown in S3 Fig. Our results show that *p*_*epitope*_ successfully distinguishes between type-specific and complex mAbs and suggests that a threshold mismatch of 20% of an epitope is sufficient to result in type-specificity. Interestingly, Gupta *et al*. found that a similar threshold of *p*_*epitope*_ = 20% corresponds to a loss of vaccine efficacy between two influenza strains [46]. For a typical mAb epitope of 25–35 residues in size, this would correspond to at least five amino acid mismatches among the epitope residues.

It is possible that antigenic variation among only a subset of epitope residues is responsible for type-specificity. For example, previous mutagenesis experiments have shown that only mutations at certain key epitope residues abrogate antibody binding, while mutations at other epitope residues have no effect [19, 24]. We extracted a subset of human mAbs in the database whose epitopes were defined exclusively by cell passaging or mutagenesis, and would thus reflect not just structural, but functionally significant epitope residues. When we generated a composite map of these epitopes on the E protein structure (S2 Fig), however, we found that the overall epitope map was similar to the epitope map using the entire database (Fig 2C), albeit more sparse. Likewise, when we looked at sequence conservation among this subset of functionally-significant epitope residues (S4 Fig), we saw similar results as when all epitope residues were considered (Fig 3A).

Finally, we compared *p*_*epitope*_ values between serotypes (inter-serotype) with *p*_*epitope*_ values within a serotype (intra-serotype), to determine if the high intra-serotype antigenic variation observed by Kaetzelnick *et al*. could be reflected in higher intra-serotype *p*_*epitope*_ values. However, as shown in S5 Fig, this was not the case. *p*_*epitope*_ values within each serotype (S5 Fig) were significantly lower than *p*_*epitope*_ values between serotypes (Fig 3B), typically below 5%. Although we sought to determine whether type-specific epitopes might have more mismatches than complex epitopes, the number of significant mismatches within a serotype (see Methods) was too small to reliably determine average *p*_*epitope*_ values. A more extensive analysis of intra-serotype variation was outside the scope of this study.

### Differences in fine-specificity between mouse and human mAbs

**Fig 4:**
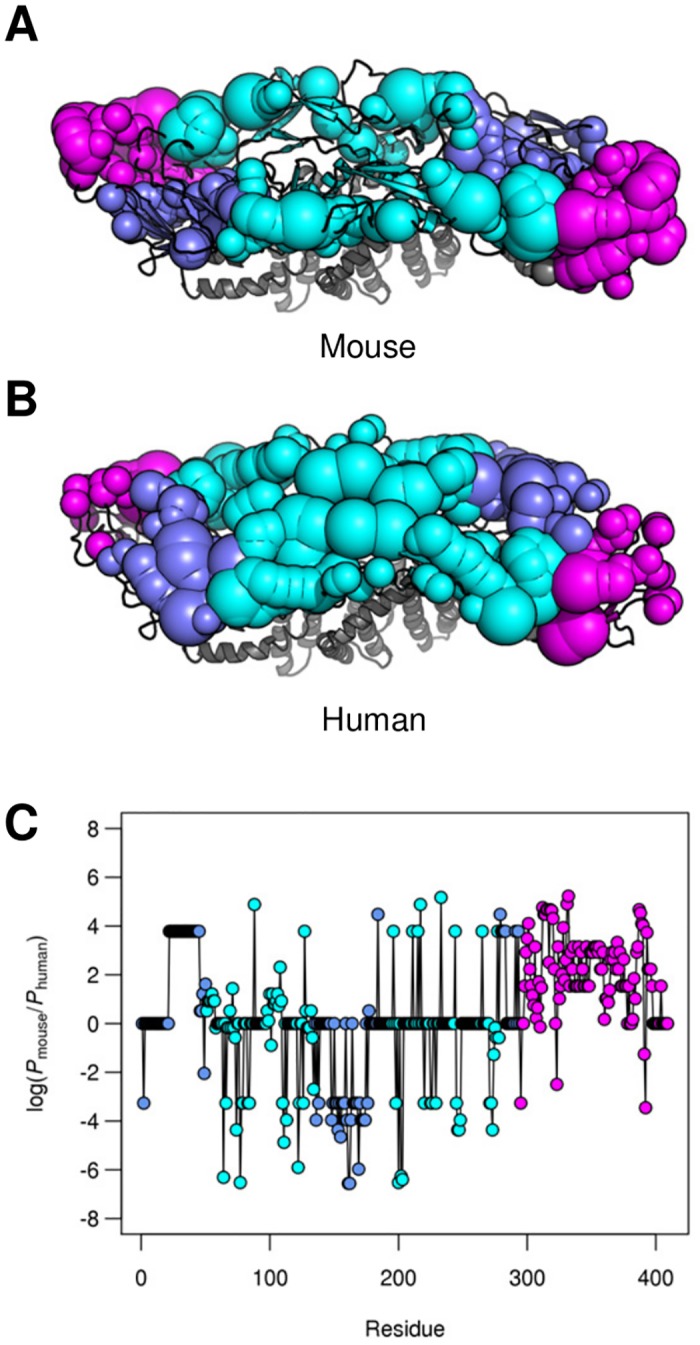
Differences in epitope fine-specificity between mouse and human. Mapping of epitope propensity on to the structure of the DENV E protein dimer for mAbs from mouse (A) and human (B). Spheres correspond to epitope residues. The size of the sphere corresponds to its epitope propensity, as described previously. C. Difference in epitope propensity between mouse and human across the E protein. Residue color corresponds to domain—purple, cyan, and magenta, for DI, DII, and DIII, respectively.

Antibody responses to DENV have been most extensively characterized in humans and in mice. We sought to determine the degree to which there are systematic differences in epitope fine-specificity between mouse and human mAbs in the dataset. Towards that end, we calculated epitope propensity for mAbs from mouse and human separately (Fig 4). Our results show that the published mAbs from mice preferentially target DIII. Whereas, published human mAbs target epitopes on the DI/DII interface and the dimer interface. These findings suggest that there are systematic differences in epitope fine-specificity between published mouse and human mAbs—in particular that the type-specific mAbs in mice predominantly target the DIII region (>75%), while published type-specific mAbs from humans target the DI/DII interface. It is important to note, however, that DIII remains a major target in the human Ab response as 30–50% of the type-specific human mAbs in our database target this region.

It is important to note that many mouse mAbs were collected in the 1990’s and 2000’s while many human mAbs were collected more recently. As such, any systematic differences in the methodologies used to select hybridomas in these studies may confound an analysis of host-level differences in the antibody responses. Indeed, 85% of human mAbs in the database were initially selected based on binding to whole-virus preparations, compared to 32% of mouse mAbs; most mouse mAbs were selected based on binding to recombinant E proteins. However, even among mouse mAbs selected based on binding to whole virus, 70% (33 of 51) targeted DIII.

## Discussion

We hypothesized that the basis for the shift in cross-reactivity in the antibody response between primary and secondary infections could be found in the epitope-level fine-specificity of the respective antibody repertoires. We based our hypothesis on the theory of original antigenic sin that during secondary infection, pre-existing memory B cells specific to cross-reactive epitopes from the primary infection, would be selectively expanded to form the B cell and antibody repertoires in secondary infection [41, 51, 52]. Our comprehensive analysis of published epitopes showed that this was partially true. For example after secondary infection, we found that Abs to type-specific DIII epitopes, such as in the DIII-lateral ridge, are significantly diminished, while Abs to cross-reactive fusion loop epitopes are increased. However, we also found that epitopes, such as the DI/DII hinge region and the dimer interface, targeted by less cross-reactive Abs following primary infection were targeted by a higher proportion of cross-reactive Abs following secondary infection. It is important to note that an epitope region encompasses many overlapping epitopes, some of which are more conserved than others. During secondary infection it is these conserved epitopes within the epitope region that appear to be selected for, increasing the apparent cross-reactivity of that region compared to the primary infection.

Overall, these results indicate that secondary DENV infections increase the number of cross-reactive Abs that target regions of the E protein recognized by both cross-reactive and type-specific Abs elicited by primary infections. This result is reminiscent of previous modeling work done in our group on a polyvalent malaria vaccine which showed that polyvalent formulations not only enhance the Ab response to shared or cross-reactive epitopes within the vaccine, but enhance the cross-reactivity of what were considered type-specific epitopes as well [53, 54]. Either serially, as in the case of secondary DENV infection, or in parallel, as in the case with polyvalent vaccine formulations, this increased cross-reactivity results from a selective advantage of cross-reactive B cells over type-specific B cells. Whether similar effects on fine-specificity and cross-reactivity are present for polyvalent dengue vaccines remains to be seen. Furthermore, the immunological consequences of the shift in epitope-level fine-specificity in the antibody response to secondary DENV infection are still unclear.

We analyzed sequence variation across DENV sequences and found that epitopes from type-specific antibodies have significantly more variation than epitopes from complex antibodies. Furthermore, we showed that the epitope-level measure, *p*_*epitope*_, successfully distinguished between type-specific and complex antibodies. In a landmark study, Katzelnick *et al*. [37] showed that even though DENV strains cluster into discrete serotypes in terms of sequence similarity along the E protein, they *do not* cluster by discrete serotypes in terms of antigenic similarity—in many cases sera raised against one serotype shows higher neutralization to a genetically distant strain from a different serotype, than to genetically similar strain from the original infecting serotype. We hypothesized that, among epitope residues identified in the database, there might be greater concordance between sequence similarity and antigenic similarity. However, this did not turn out to be the case. When we calculated *p*_*epitope*_ values across the 47 DENV strains tested in that study, we found that intra-serotype *p*_*epitope*_ values were relatively low (<5%) and far exceeded by inter-serotype *p*_*epitope*_ values, for epitopes from both type-specific and complex antibodies. Previous studies have found that a small number of mutations can result in significant strain-specific differences in neutralization [27, 39, 55], that sequence variation alone failed to predict strain-specific differences in neutralization [56], and that genotype differences in viral conformational dynamics and epitope accessibility may be responsible [57]. As of yet, the structural and immunological basis for why DENV strains do not cluster antigenically into discrete serotypes is still largely unknown.

Many previously published type-specific mouse mAbs predominantly target DIII of the E protein, while a lower proportion of characterized human mAbs (30–50% in the database) target this region [30, 31, 58]. Furthermore, type-specific human Abs predominately target other E protein regions such as the DI/DII hinge region that have not been described for mouse mAbs. Our findings suggest this might be the result of systematic differences in epitope fine specificity between the mouse and human antibody responses but may also reflect differences in experimental methods used to produce mAbs from mice versus humans.

We developed the DENV antibody database to provide a repository for activity and epitope information for DENV-specific mAbs in order to better characterize repertoire-level properties of the DENV antibody response. Here we provide an overview of the database and demonstrate how it can be used to analyze the relationship between epitope fine-specificity and serotype cross-reactivity in primary and secondary infections. We invite readers to explore the DENV antibody database (http://denvabdb.bhsai.org), use it both as repository for storing and accessing information on DENV mAbs, and as a means to systematically analyze and characterize DENV antibody responses.

## Supporting information

S1 Fig Sequence diversity in DENV. Sequence diversity (1 − *sequence conservation*) was calculated from 47 DENV strains and is shown for DENV-1, DENV-2, DENV-3, and DENV-4 serotypes, across the E protein.
(TIF)

S2 Fig Composite epitope map in primary and secondary infection. Composite epitope maps that were generated from epitopes defined exclusively from mutagenesis or cell passaging experiments from human mAbs from primary and secondary infections. Spheres correspond to epitope residues. The size of the sphere corresponds to its epitope propensity: low propensity (<5%, small spheres), medium propensity (>5% and <10%; medium spheres), and high propensity (>10%, large spheres). The color of the sphere corresponds to the epitope cross-reactivity as described above.
(TIF)

S3 Fig Distribution of epitope definition sizes used in the *p*_*epitope*_ calculations. Histogram showing the the percentage of antibodies in the data set that have a poorly defined (5–10 residues), moderately well defined (10–20 residues), and well-defined epitopes (20+) in the data set.
(TIF)

S4 Fig Sequence variation and serotype specificity in DENV mAbs. Histogram of sequence conservation among DENV 1–4 E protein for epitope residues defined exclusively by cell passaging and mutagenesis experiments, for type-specific and complex human mAbs from primary and secondary infections.
(TIF)

S5 Fig Strain-level sequence variation within serotypes. Average and standard deviation of pairwise *p*_*epitope*_ values within each serotype for primary type-specific mAbs, primary complex-specific mAbs, and secondary, complex-specific mAbs. Y-axis is scaled to the same range as Fig 3 and S4 Fig for comparison.
(TIF)

S1 Table Sequences used to calculate antigenic variation. (PDF)

S2 Table Human mAb origin and selection information. (XLSX)
