# Supplementary figures and images for: Dengue virus antibody database: Systematically linking serotype-specificity with epitope mapping in dengue virus
Source: PLoS Negl Trop Dis. 2017 Feb 21;11(2):e0005395. doi: 10.1371/journal.pntd.0005395 (PMC5336305; doi:10.1371/journal.pntd.0005395)

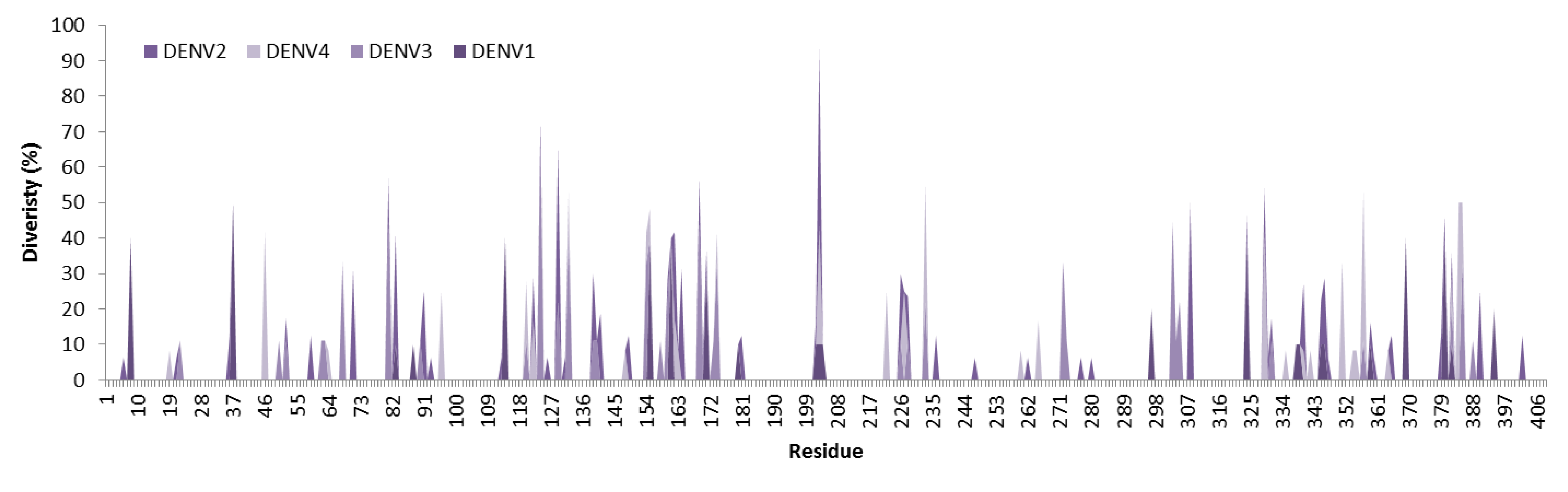

Supplement: S1 Fig — Sequence diversity (1 − sequence conservation) was calculated from 47 DENV strains and is shown for DENV-1, DENV-2, DENV-3, and DENV-4 serotypes, across the E protein. (TIF) [file pntd.0005395.s001.tif]

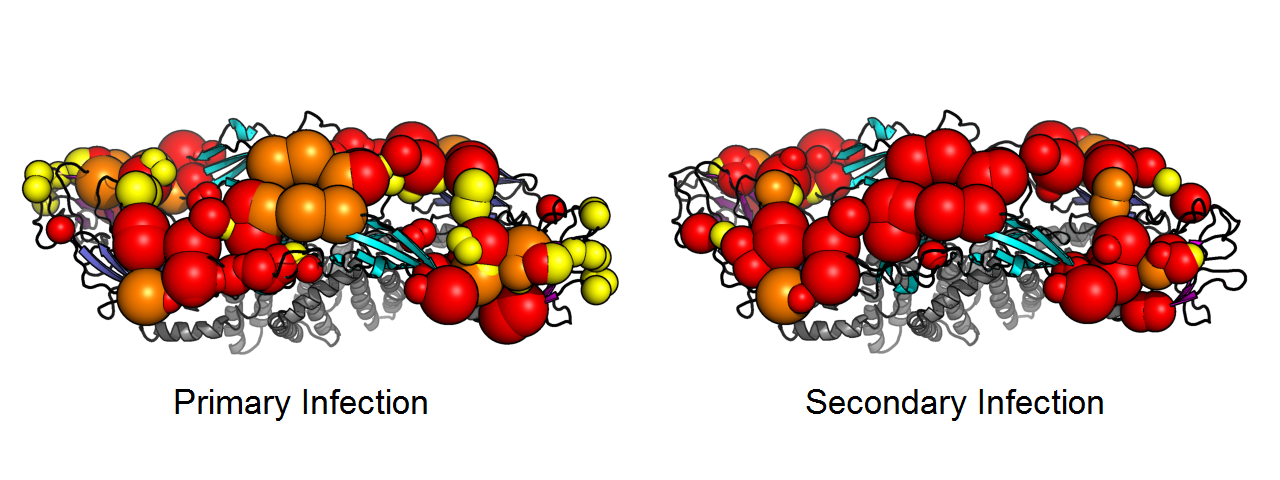

Supplement: S2 Fig — Composite epitope maps that were generated from epitopes defined exclusively from mutagenesis or cell passaging experiments from human mAbs from primary and secondary infections. Spheres correspond to epitope residues. The size of the sphere corresponds to its epitope propensity: low propensity (<5%, small spheres), medium propensity (>5% and <10%; medium spheres), and high propensity (>10%, large spheres). The color of the sphere corresponds to the epitope cross-reactivity as described above. (TIF) [file pntd.0005395.s002.tif]

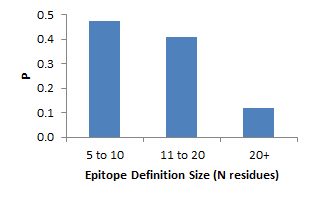

Supplement: S3 Fig — Histogram showing the the percentage of antibodies in the data set that have a poorly defined (5–10 residues), moderately well defined (10–20 residues), and well-defined epitopes (20+) in the data set. (TIF) [file pntd.0005395.s003.tif]

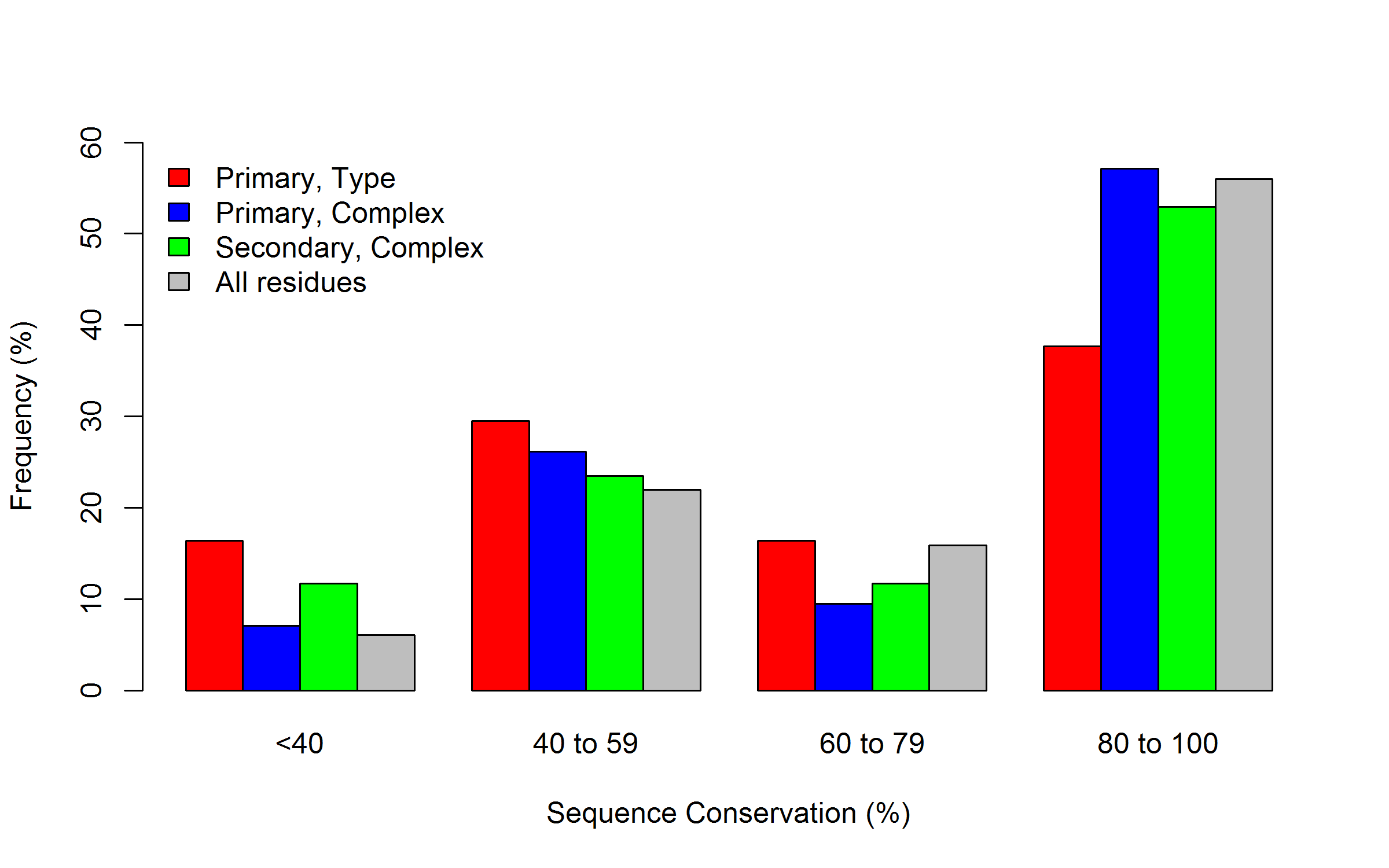

Supplement: S4 Fig — Histogram of sequence conservation among DENV 1–4 E protein for epitope residues defined exclusively by cell passaging and mutagenesis experiments, for type-specific and complex human mAbs from primary and secondary infections. (TIF) [file pntd.0005395.s004.tif]

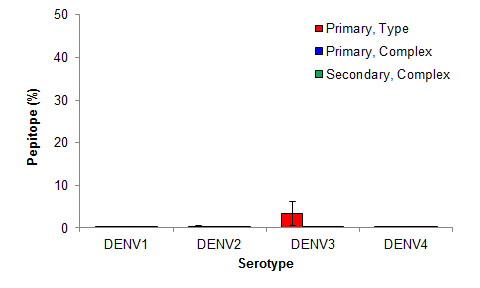

Supplement: S5 Fig — Average and standard deviation of pairwise pepitope values within each serotype for primary type-specific mAbs, primary complex-specific mAbs, and secondary, complex-specific mAbs. Y-axis is scaled to the same range as Fig 3 and S4 Fig for comparison. (TIF) [file pntd.0005395.s005.tif]
