# Supplementary material for: Dengue virus antibody database: Systematically linking serotype-specificity with epitope mapping in dengue virus
Source: PLoS Negl Trop Dis. 2017 Feb 21;11(2):e0005395. doi: 10.1371/journal.pntd.0005395 (PMC5336305; doi:10.1371/journal.pntd.0005395)
Supplement: S1 Table — (PDF) [file pntd.0005395.s006.pdf]

Name	GenBank
DENV2/India/1974-Poona-742295	FJ538920
DENV2/NewGuinea/1944-NewGuineaC	AF038403
DENV2/Vietnam/2006-32-135	EU482672
DENV2/Nicaragua/2006-BID-V571	EU482684
DENV2/Nicaragua/2005-BID-V533	EU482756
DENV2/Brazil/2004-BR-161	KT382189
DENV2/Guyana/2000-CAREC-00-08221	KT382188
DENV2/Vietnam/2003-AC21	KT452796
DENV2/Vietnam/2003/DF670-AC20	KT452797
DENV2/Cambodia/2008-BID-V3924	GQ868638
DENV2/Cambodia/2009/D2T0601085_KH09_KSP	KT452795
DENV2/Cambodia/2007/BID-V4265	GU131927
DENV2/Peru/1996-IQT-2913	AY158339
DENV2/Tonga/1974-Tonga/74	AY744147
DENV2/Malaysia/2008-DKD-811	FJ467493
DENV2/Senegal/1970/Sendak_H	EF105384
DENV3/Indonesia/1978/Sleman-1280-AC25	KT452798
DENV3/Vietnam/2006-BID-V1329	EU660409
DENV3/Vietnam/2007-BID-V1817	FJ432743
DENV3/Myanmar/2008/80931	KT452792
DENV3/Cambodia/2011/V0907330-AC23	KT452799
DENV3/Fiji/1992-29472-L11422-I	L11422
DENV3/PuertoRico/2006-429965	EU529698
DENV3/Nicaragua/2009-BID-V4753	HQ541806
DENV3/Puerto-Rico/1963/PRS-228762-AC27	KT452800
DENV1/Thailand/1964-16007	AF180817
DENV1/Nauru/1974-WestPac	AY145121
DENV1/PuertoRico/2006-BID-V852	EU482591
DENV1/Bolivia/2010-FSB-3363	KT382187
DENV1/Venezuela/2000-OBT-1298	KT382186
DENV1/Peru/2000-IQT-6152	AY780643
DENV1/Vietnam/2008-BID-V1937	FJ461335
DENV1/Cambodia/2003-BID-V1995	FJ639680
DENV1/Cambodia/2003-BID-V1991	GQ868619
DENV1/Myanmar/2005/61117	KT452791
DENV4/Indonesia/1973/M30153-AC36	KT452801
DENV4/Indonesia/1978-S1228	JN022608
DENV4/Dominica/1981-42A	AF326573
DENV4/PuertoRico/1998-347751	EU854297
DENV4/PuertoRico/1999-BID-V2446	FJ882599
DENV4/Nicaragua/1999-703	KT452803
DENV4/Brazil/2012/BR-12	KT452794
DENV4/Thailand/1985-D85-052	AY780644
DENV4/Cambodia/2010-U0811386	KF543272
DENV4/Cambodia/2011/V0624301-AC33	KT452802
DENV4/Myanmar/2008/81087	KT452793
DENV4/Malaysia/1973/P73-1120	JF262780
